# Supplementary material for: Multimodal MRI improves diagnostic accuracy and sensitivity to longitudinal change in amyotrophic lateral sclerosis
Source: Commun Med (Lond). 2023 Jun 16;3:84. doi: 10.1038/s43856-023-00318-5 (PMC10276031; doi:10.1038/s43856-023-00318-5)
Supplement: Supplementary file 1 — Description of Additional Supplementary Files [file 43856_2023_318_MOESM1_ESM.pdf]

# Description of Additional Supplementary File

**File Name:** Supplementary Data 1

**Description:** Volume and MNI coordinates of affected regions

**File Name:** Supplementary Data 2

**Description:** The source data underlying Figures 2, 3, and 6
