# Supplementary material for: Multimodal MRI improves diagnostic accuracy and sensitivity to longitudinal change in amyotrophic lateral sclerosis
Source: Commun Med (Lond). 2023 Jun 16;3:84. doi: 10.1038/s43856-023-00318-5 (PMC10276031; doi:10.1038/s43856-023-00318-5)
Supplement: Supplementary file 2 — Supplementary information [file 43856_2023_318_MOESM2_ESM.pdf]

Supplementary information: Improved Diagnostic Accuracy and Sensitivity to Longitudinal Change in ALS with Brain and Cervical Cord MRI

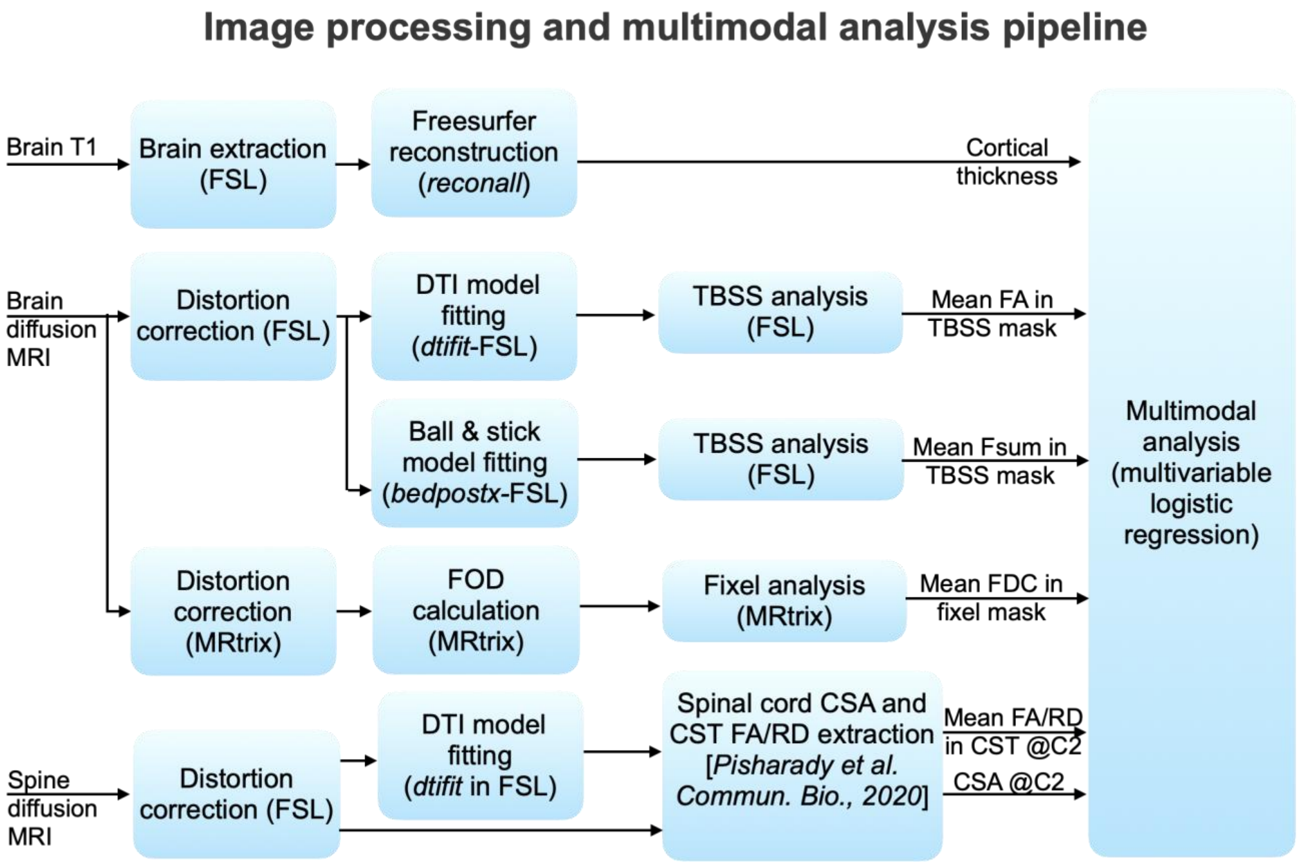

Supplementary figure 1. Flow diagram depicting the image processing and multimodal analysis pipeline (DTI - diffusion tensor imaging, TBSS - tract-based spatial statistics, FA - fractional anisotropy, RD - radial diffusivity, Fsum - sum of fiber volume fractions, FOD - fiber orientation distribution, FDC - fiber density and cross-section, CST - corticospinal tract, CSA - cross-sectional area).

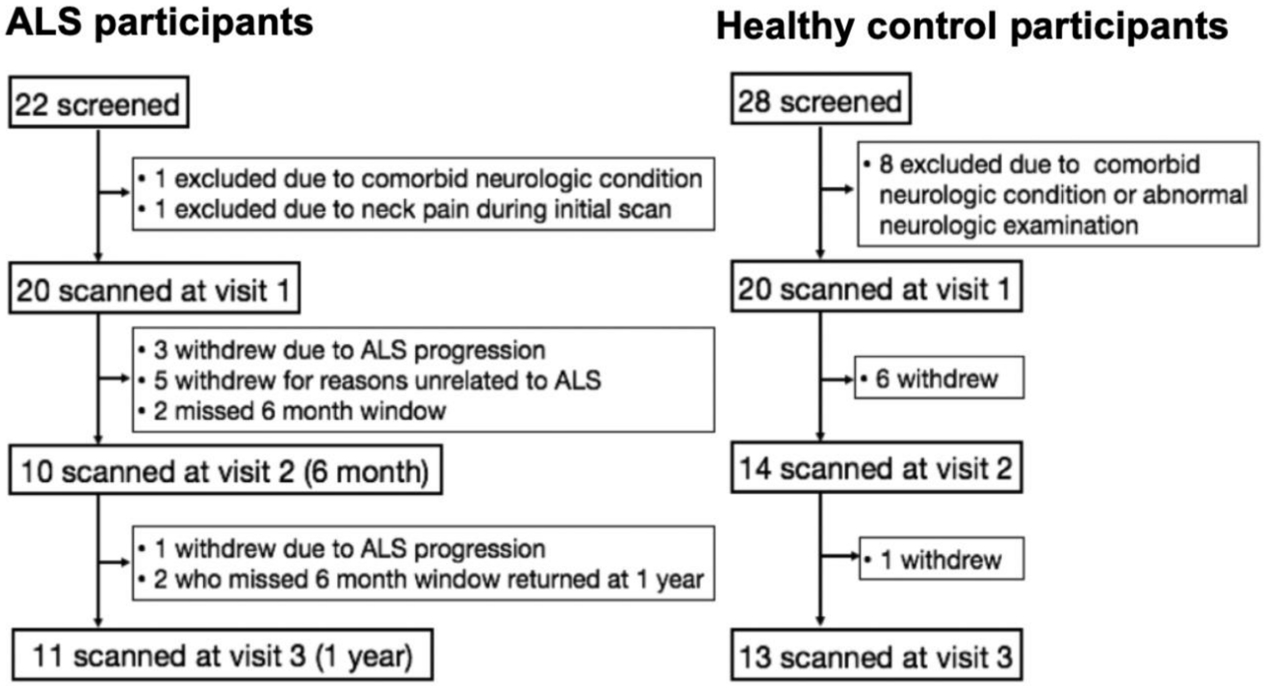

Supplementary figure 2. Flow diagram of study participants. ALS participant withdrawals were recorded as being either due to ALS progression or due to reasons unrelated to ALS.

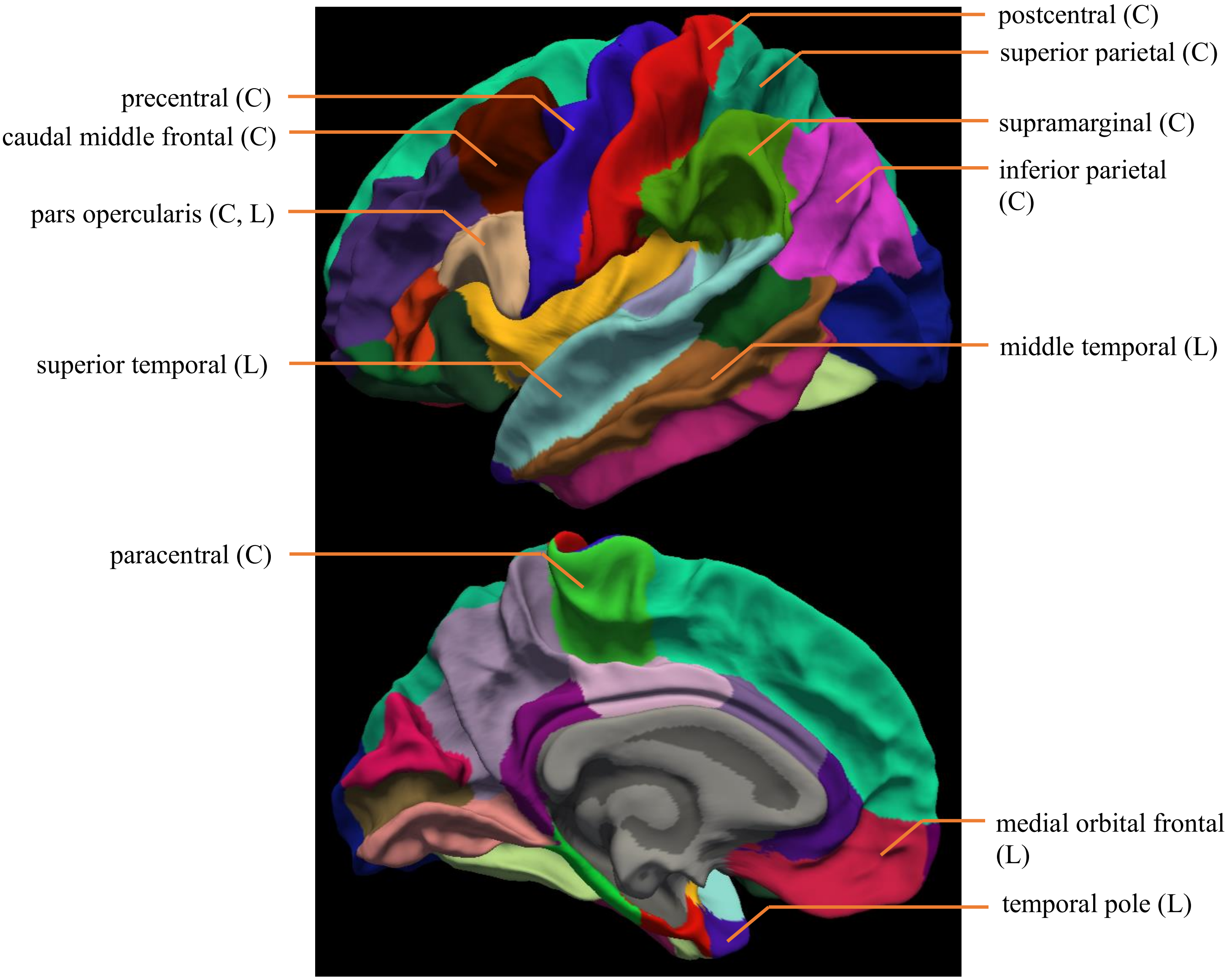

Supplementary figure 3. Cortical regions from the Desikan–Killiany atlas[26] with the regions labeled where cortical thinning is noted. The letters C and L in parenthesis represent regions where cross-sectional difference and longitudinal change (respectively) are noted in the t-test.

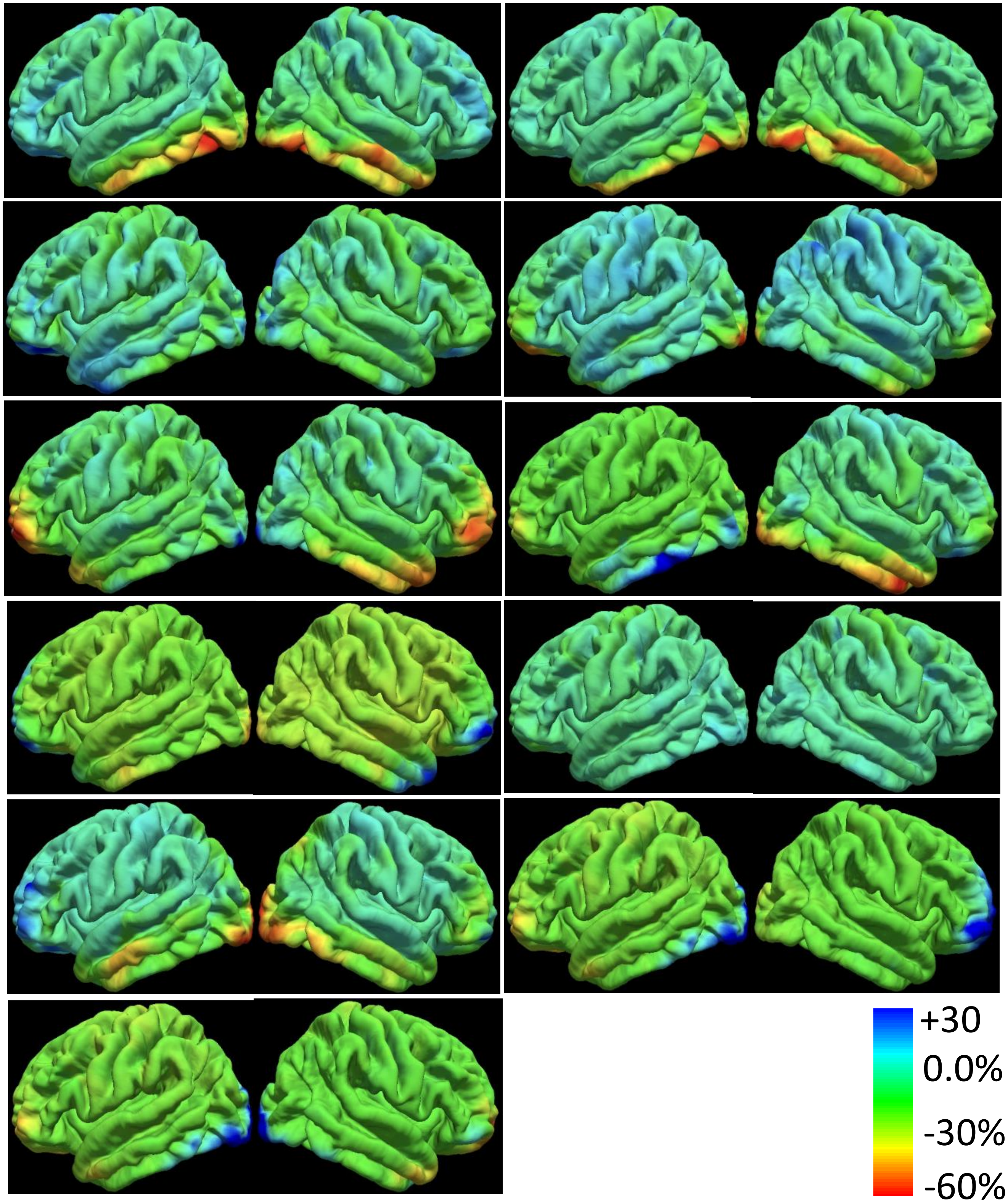

Supplementary figure 4. Left and right views of longitudinal change (baseline vs. 12-month) in cortical thickness in the 11 ALS participants. The change is expressed as a percentage of the cortical thickness at the baseline. Negative sign represents cortical thinning over 12 months, and the negative values convey the percentage thinning at a given cortical location.

Supplementary Table 1 ALS vs. control group classification performance based on multimodal longitudinal 12-month change data.

Measure	Accuracy (%)	Sensitivity (%)	Specificity (%)
**Brain FA**	**87.5**	**81.8**	**92.3**
Brain Fsum	83.3	81.8	84.6
Superior temporal cortical thickness	54.2	45.5	61.5
Spinal cord RD CST at C2	66.7	45.5	84.6
Spinal cord CSA at C2	41.7	18.2	61.5
Brain FDC	75.0	72.7	77.0
Multimodal*	79.2	81.8	77.0

*Multimodal analysis included brain FA, cortical thickness, spinal cord CST RD at C2, spinal cord CSA at C2, and brain FDC (ALS - amyotrophic lateral sclerosis, FA - fractional anisotropy, Fsum - sum of fiber volume fractions, CST - corticospinal tract, C2 - spinal cord C2 level, CSA - cross-sectional area, RD - radial diffusivity, FDC - fiber density and cross-section). Brain FA (in bold) provided the best classification accuracy.
