# Supplementary material for: Multimodal MRI improves diagnostic accuracy and sensitivity to longitudinal change in amyotrophic lateral sclerosis
Source: Commun Med (Lond). 2023 Jun 16;3:84. doi: 10.1038/s43856-023-00318-5 (PMC10276031; doi:10.1038/s43856-023-00318-5)
Supplement: Supplementary file 6 — Reporting Summary [file 43856_2023_318_MOESM6_ESM.pdf]

nature portfolio

Corresponding author(s): Pramod K Pisharady

Last updated by author(s): May 15, 2023

Reporting Summary

Nature Portfolio wishes to improve the reproducibility of the work that we publish. This form provides structure for consistency and transparency in reporting. For further information on Nature Portfolio policies, see our Editorial Policies and the Editorial Policy Checklist.

Statistics

For all statistical analyses, confirm that the following items are present in the figure legend, table legend, main text, or Methods section.

n/a	Confirmed	
☐	☒	The exact sample size (*n*) for each experimental group/condition, given as a discrete number and unit of measurement
☐	☒	A statement on whether measurements were taken from distinct samples or whether the same sample was measured repeatedly
☐	☒	The statistical test(s) used AND whether they are one- or two-sided<br>*Only common tests should be described solely by name; describe more complex techniques in the Methods section.*
☐	☒	A description of all covariates tested
☐	☒	A description of any assumptions or corrections, such as tests of normality and adjustment for multiple comparisons
☐	☒	A full description of the statistical parameters including central tendency (e.g. means) or other basic estimates (e.g. regression coefficient) AND variation (e.g. standard deviation) or associated estimates of uncertainty (e.g. confidence intervals)
☐	☒	For null hypothesis testing, the test statistic (e.g. *F*, *t*, *r*) with confidence intervals, effect sizes, degrees of freedom and *P* value noted<br>*Give P values as exact values whenever suitable.*
☒	☐	For Bayesian analysis, information on the choice of priors and Markov chain Monte Carlo settings
☒	☐	For hierarchical and complex designs, identification of the appropriate level for tests and full reporting of outcomes
☐	☒	Estimates of effect sizes (e.g. Cohen's *d*, Pearson's *r*), indicating how they were calculated

Our web collection on statistics for biologists contains articles on many of the points above.

Software and code

Policy information about availability of computer code

Data collection	Data acquisition softwares were supplied with Siemens Trio and Prisma scanners
Data analysis	Our code is developed in Matlab software, we used MATLAB 9.10 (R2021a) for running the code.<br>We also used FSL version 6.0.3 and MRtrix3 (2019) softwares for the analysis.

For manuscripts utilizing custom algorithms or software that are central to the research but not yet described in published literature, software must be made available to editors and reviewers. We strongly encourage code deposition in a community repository (e.g. GitHub). See the Nature Portfolio guidelines for submitting code & software for further information.

Data

Policy information about availability of data

All manuscripts must include a data availability statement. This statement should provide the following information, where applicable:

- Accession codes, unique identifiers, or web links for publicly available datasets
- A description of any restrictions on data availability
- For clinical datasets or third party data, please ensure that the statement adheres to our policy

Data availability:
Deidentified data will be made available on request for the purposes of reproducing the results presented, subject to institutional approval. The source data underlying Figures 2, 3, and 6 are made available as Supplementary Data 2.

March 2021

Code availability:
The code used to produce the results reported in this article is available at https://www.cmrr.umn.edu/downloads/alsmultimodal/index.php

Human research participants

Policy information about studies involving human research participants and Sex and Gender in Research.

Reporting on sex and gender	Our findings are based on both genders, we recruited people of both sex in our cohort to minimize the effect of gender. The baseline sex ratio were (M:F) 11:9 for ALS and 10:10 for controls. The sex ratio at one-year follow-up were (M:F) 7:4 for ALS and 6:7 for controls.
Population characteristics	The ALS cohort was on average an early-stage cohort with mean ALSFRS-R of 40.0, with 60% in King's Stage 1 or 2 at time of enrollment. This proportion was reduced to 45.5% at the one-year follow-up visit. As per revised El Escorial Criteria, there were 7 possible, 8 probable, and 5 definite subjects at baseline. Out of the 7 possible subjects, 1 progressed to definite, 2 progressed to probable, 3 remained as possible, and 1 was withdrawn from the study at the one-year follow-up. Out of the 8 probable subjects, 3 remained as probable and 5 were withdrawn from the study. Only two out of the five definite subjects returned for the one-year follow-up and they remained in the definite status. The mean change in ALSFRS-R of ALS participants at the one-year follow-up visit was -5.0 points, with an average slope of -0.4 points/month. There were four deaths in the ALS cohort and no deaths in the control cohort during the study.
Recruitment	People who met revised El Escorial Criteria for clinically possible, probable, or definite ALS were recruited from the ALS Association Certified Treatment Centers of Excellence at the University of Minnesota and Hennepin County Medical Center. Healthy control volunteers were recruited from the general public and selected to match ALS participants' age range and sex frequency. Exclusion criteria included the presence of neurologic illnesses other than ALS, the inability to tolerate MRI scanning, and the failure to meet MRI safety criteria.
Ethics oversight	Institutional Review Board, University of Minnesota

Note that full information on the approval of the study protocol must also be provided in the manuscript.

Field-specific reporting

Please select the one below that is the best fit for your research. If you are not sure, read the appropriate sections before making your selection.

☒ Life sciences ☐ Behavioural & social sciences ☐ Ecological, evolutionary & environmental sciences

For a reference copy of the document with all sections, see nature.com/documents/nr-reporting-summary-flat.pdf

Life sciences study design

All studies must disclose on these points even when the disclosure is negative.

Sample size	We did not do a sample size calculation as there is no straightforward sample size calculation formula for the multi modal analysis we conducted. Also, as there is no such prior multimodal data available, a simulation study would need to be designed and carried out to obtain an approximate sample size. Such simulation studies typically perform best when there is some pilot data available, on which to base them. We could perform a standard sample size calculation post-hoc for progression of single modality data, but this will not show the statistical power of the multimodal method we used.
Data exclusions	We did not exclude any acquired data from the analysis.
Replication	The results and findings we report can be reproduced as they are obtained using computer code operating on MRI data. However, we have not included "test-retest" reproducibility data [NOTE: we have this data from our another study on FRDA which shared the same protocol].
Randomization	This is a group analysis comparing patients with healthy controls. People who met revised El Escorial Criteria for clinically possible, probable, or definite ALS were recruited to the patient population. Healthy volunteers were recruited as controls from the general public and selected to match ALS participants' age range and sex frequency.
Blinding	The disease status of the subjects was known to the investigators as the objective was to find the differences between the two groups (patients and controls), given their status. In order to report the accuracy, sensitivity, and specificity of the diagnosis, we did leave-one-out cross-validation. We gave all the subject labels together as input to the algorithm and the computer automatically tested them one-by-one without manual intervention.

Reporting for specific materials, systems and methods

We require information from authors about some types of materials, experimental systems and methods used in many studies. Here, indicate whether each material, system or method listed is relevant to your study. If you are not sure if a list item applies to your research, read the appropriate section before selecting a response.

Materials & experimental systems

n/a	Involved in the study
☒	☐ Antibodies
☒	☐ Eukaryotic cell lines
☒	☐ Palaeontology and archaeology
☒	☐ Animals and other organisms
☒	☐ Clinical data
☒	☐ Dual use research of concern

Methods

n/a	Involved in the study
☒	☐ ChIP-seq
☒	☐ Flow cytometry
☐	☒ MRI-based neuroimaging

Magnetic resonance imaging

Experimental design

Design type: Microstructural analysis using diffusion MRI

Design specifications: Subjects were scanned at baseline, 6 months, and 12 months.

Behavioral performance measures: There were no performance measures during the MRI data acquisition. The functional impairment in ALS participants was measured using the ALS Functional Rating Scale-Revised (ALSFRS-R), before the MRI data acquisition. Cognitive and behavioral status was also assessed using the Edinburgh Cognitive Behavioral ALS Screen (ECAS).

Acquisition

Imaging type(s): T1 and diffusion MRI

Field strength: 3T

Sequence & imaging parameters: Diffusion encoding was applied along 128 directions with a b-value of 1500 s/mm2 (TR=4253 ms, TE=90.6 ms). Seventeen additional volumes without diffusion encoding were equally interleaved in the dataset yielding a total of 145 volumes. We obtained 90 slices with thickness 1.8 mm and voxel size 1.81x1.81 mm2 (FoV=106x106). Two sets of data were collected during each session, with reversed phase encoding directions (anterior to posterior and posterior to anterior). T1 images are acquired at an isometric resolution of 1 mm with a size of 176x256x224 voxels (TR=2530 ms, TE=3.65 ms).

Area of acquisition: Whole brain

Diffusion MRI: ☒ Used ☐ Not used

Parameters: 128 diffusion directions, b-value 1500 s/mm2, Seventeen b0 volumes, 90 slices with thickness 1.8 mm and voxel size 1.81x1.81 mm2, TR=4253 ms, TE=90.6 ms.

Preprocessing

Preprocessing software: We used FSL version 6.0.3, MRtrix3 (2019), and MATLAB 9.10 (R2021a).

Normalization: Particular normalization of the data was not required (except for the below structural registration) as the features are extracted after diffusion tensor model (DTI) fitting.

Normalization template: We used templates created from our cohort.

Noise and artifact removal: The data were corrected for distortions due to eddy currents, susceptibility-induced off-resonance artifacts and subject motion using FSL version 6.0.3.

Volume censoring: No volume censoring was done

Statistical modeling & inference

Model type and settings: The multimodal analysis was done using multivariable logistic regression. Leave-one-out cross-validation was done to report the mean accuracy, sensitivity, and specificity (further details are provided in the statistical analysis section of the paper).

Effect(s) tested: The effect of ALS disease on microstructural and morphological features of the brain

Specify type of analysis: ☐ Whole brain ☐ ROI-based ☒ Both

Anatomical location(s): We first conducted the whole brain analysis and then did the ROI analysis of regions where disease effect is detected (by the computer algorithm).

Statistic type for inference<br>(See Eklund et al. 2016)	We report both voxel-wise and cluster-wise results
Correction	The p-values were corrected for multiple testing (across the multiple modalities) using the Bonferroni-Holm method (see reference 25 in the manuscript for details).

Models & analysis

n/a	Involved in the study
☒	☐ Functional and/or effective connectivity
☒	☐ Graph analysis
☐	☒ Multivariate modeling or predictive analysis

Multivariate modeling and predictive analysis	The independent variables were the MRI-based diffusion and morphology metrics from the brain and the spinal cord. The disease status (patient vs. control) was the response variable.
